# TimerQuant: a modelling approach to tandem fluorescent timer design and data interpretation for measuring protein turnover in embryos

**DOI:** 10.1242/dev.125971

**Published:** 2016-01-01

**Authors:** Joseph D. Barry, Erika Donà, Darren Gilmour, Wolfgang Huber

**Affiliations:** EMBL Heidelberg, Meyerhofstrasse 1, Heidelberg 69117, Germany

**Keywords:** Protein turnover, Fluorescent timer, Mathematical modelling

## Abstract

Studies on signalling dynamics in living embryos have been limited by a scarcity of *in vivo* reporters. Tandem fluorescent protein timers provide a generic method for detecting changes in protein population age and thus provide readouts for signalling events that lead to changes in protein stability or location. When imaged with quantitative dual-colour fluorescence microscopy, tandem timers offer detailed ‘snapshot’ readouts of signalling activity from subcellular to organismal scales, and therefore have the potential to revolutionise studies in developing embryos. Here we use computer modelling and embryo experiments to explore the behaviour of tandem timers in developing systems. We present a mathematical model of timer kinetics and provide software tools that will allow experimentalists to select the most appropriate timer designs for their biological question, and guide interpretation of the obtained readouts. Through the generation of a series of novel zebrafish reporter lines, we confirm experimentally that our quantitative model can accurately predict different timer responses in developing embryos and explain some less expected findings. For example, increasing the FRET efficiency of a tandem timer actually increases the ability of the timer to detect differences in protein half-life. Finally, while previous studies have used timers to monitor changes in protein turnover, our model shows that timers can also be used to facilitate the monitoring of gene expression kinetics *in vivo*.

## Abstract (precis)

**Summary:** A quantitative dual-colour reporter system can be used to measure protein turnover, signalling activity and gene expression kinetics in living zebrafish embryos.

## INTRODUCTION

Recent advances in microscopy technologies have allowed cell and tissue behaviours in living embryos to be visualised at unprecedented temporal and spatial resolution (Keller, 2013; Pantazis and Supatto, 2014). Although the identity of key molecular players orchestrating such behaviours is often known, their activity dynamics remain poorly understood. Progress in this direction has been limited by a general lack of probes that allow protein activity to be monitored in living tissues. As many biochemical activities are known to result in the stabilisation, destruction or relocalisation of target proteins (Stamos and Weis, 2013; Zhao et al., 2010), fluorescent timer approaches that allow measurement of protein population age *in vivo* provide a potentially generic solution to this problem.

We have recently employed tandem fluorescent protein timers, originally developed to address protein turnover in *S. cerevisiae* (Khmelinskii et al., 2012), to study chemokine signalling and cadherin stability along the zebrafish posterior lateral line primordium (Donà et al., 2013; Revenu et al., 2014), a model system for the study of collective migration and organ assembly during development. Tagging the receptor Cxcr4b with a tandem timer revealed a gradient of chemokine activity across the tissue and showed that receptor lifetimes respond rapidly to acute changes in chemokine level. These, and other experiments, demonstrate that tandem timers are well suited for studies in living embryos. Comprising two spectrally distinguishable fluorescent proteins with different maturation rates, tandem timers combine the advantages of protein tagging with single fluorescent proteins, such as visualisation of protein abundance and localisation, with a quantitative measurement of protein population age obtained from the fluorescence intensity ratio between the slow- and fast-maturing fluorophore channels. This ‘timer ratio’ measurement of protein lifetime is independent of production rate, a great advantage for *in vivo* contexts where tightly defined reporter expression is not practical. A further advantage is the snapshot capability of the tandem timer. Since a single time point measurement is sufficient to form the timer ratio, imaging throughput is greatly enhanced. Additionally, because users are free to select the fluorophores on the tag, maturation rates can be tuned to be appropriate for the half-lives of the proteins under study.

The broad range of developmental contexts in which timer approaches could provide insight calls for a theoretical framework in which quantitative predictions about timer behaviours can be made. We present a mathematical model of timer kinetics and associated software tools that allow users to interpret timer readouts and to detect differences in protein half-life (http://chronos.embl.de/TimerQuant). We validate model predictions by applying a set of timers to investigate chemokine signalling gradients across the zebrafish posterior lateral line primordium. Finally, using simulations that distinguish between production and degradation dynamics, we show that the tandem timer approach not only reports on protein turnover, but can also be applied to track dynamic changes in gene expression, a process of particular importance in developmental systems.

## RESULTS AND DISCUSSION

### Using a mathematical model to quantify timer signal

**Fig. 1.**
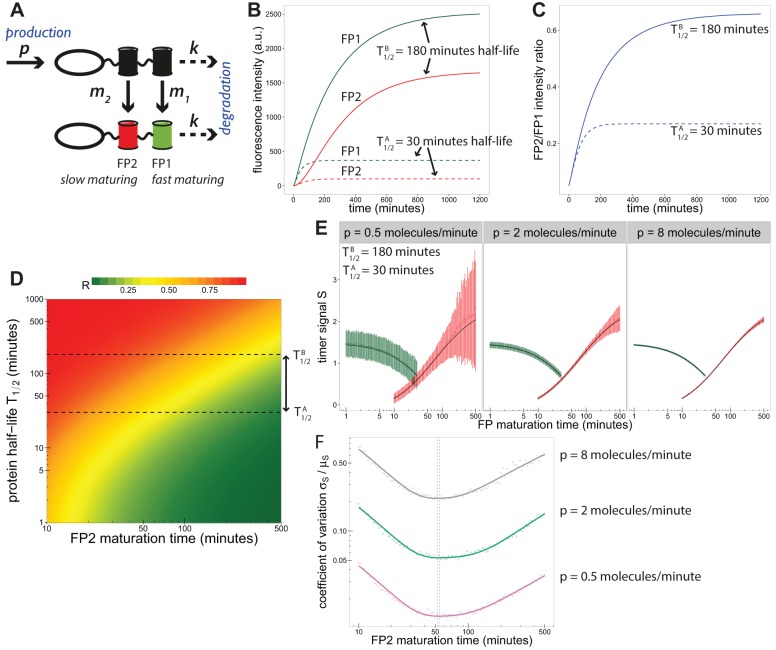
**A mathematical model allows for quantification of timer signal.** (A) Schematic of model used to describe timer behaviour. (B) Model solutions for fluorophore intensities over time. Solid and dashed lines show solutions for proteins with half-lives of 180 and 30 min, respectively. (C) FP2/FP1 fluorescence intensity ratios over time, showing a higher ratio for the longer-lived protein. (D) Steady-state ratios (*R*) as a function of FP2 maturation time and protein half-life (*t*_1_=5 min). Dashed lines highlight ratios for the protein half-lives under inspection in B,C. (E) Results of computer simulations with an additive error model. Green profiles show signal decreasing with FP1 maturation time (*t*_2_=100 min). Red profiles show signal increasing with FP2 maturation time (*t*_1_=5 min). Error bars indicate s.d. Solid lines show model solutions without additive noise. Panels show results for different protein production rates (*p*). (F) The coefficient of variation is used to predict an optimal FP2 maturation time (*t*_1_=5 min). Solid lines are fits to the data, from which a local minimum was calculated (vertical dashed lines).

To investigate tandem timer behaviour we used an ordinary differential equation model that models the production, degradation and fluorophore maturation kinetics of a pool of proteins tagged with the timer (Khmelinskii et al., 2012). We denote the faster maturing fluorescent protein as FP1 [maturation rate *m*_1_, maturation time *t*_1_=log(2)/*m*_1_] and the slower maturing fluorescent protein as FP2 [maturation rate *m*_2_, maturation time *t*_2_=log(2)/*m*_2_]. Proteins are produced at a constant rate *p* and degraded at a constant rate *k* (Fig. 1A). Model solutions show fluorescence intensities smoothly increasing over time before approaching a limit where intensity no longer changes over time, a condition that we refer to as a steady state (Fig. 1B). Owing to the faster maturation kinetics of FP1, the fraction of the protein pool that becomes fluorescent before being removed by degradation is higher for FP1 than for FP2 (Fig. 1B). As protein half-life increases, more FP2 proteins mature to fluorescence before being degraded, resulting in a higher FP2/FP1 intensity ratio (Fig. 1B,C). Protein half-life *T*_1/2_ and degradation rate *k* are related by *T*_1/2_=log(2)/*k*.

Tandem timers can be made with virtually any combination of spectrally distinguishable FPs, each characterised by a specific maturation time. Since the existing FP catalogue offers a wide range of FPs for timer design, this raises the question of which fluorophore combination to choose. We used the model to determine how the fluorophore pair choice affects the ability of a timer to detect changes in protein half-life. We first modelled steady-state ratios for different choices of FP2 maturation time while keeping FP1 maturation time fixed (Fig. 1D). We inspected timer ratios for two protein half-lives, 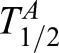
=30 min and 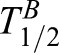
=180 min (Fig. 1D, dashed lines), and noticed that the corresponding change in the timer ratios varied considerably with FP2 maturation time.

Next, we defined timer signal *S* as the log_2_ fold-change between the steady-state ratios of the longer-lived and shorter-lived proteins: log_2_(*R^B^*/*R^A^*). To account for unavoidable measurement noise we used an additive noise model in which random variables drawn from a normal distribution with a fixed variance σ^2^ were added to each fluorescence channel (see Mathematical methods in the Materials and Methods). Thus, the data summary statistic *S* was modelled as a random variable with mean *μ_S_* and standard deviation *σ_S_*, which we estimated by computer simulation (Fig. 1E). Timer signal (*μ_S_*) decreased with FP1 maturation time, indicating that it is advisable to choose the fastest available FP1 fluorophore. Conversely, both timer signal and variability (*σ_S_*) increased with FP2 maturation time, which is expected because FP2 intensity tends to zero as its maturation time increases, making the effect of background noise more pronounced. We repeated the simulations for different protein production rates, and found that variability decreases with protein abundance (Fig. 1E). Thus, a slow-maturing FP2 is preferable for highly abundant proteins since the model predicts high signal while variability remains low.

Next, we inspected the coefficient of variation (CV, *σ_S_*/*μ_S_*) and found that, desirably, the profiles attain a minimum value that is independent of protein abundance (Fig. 1F), suggesting that the model can predict an optimal FP2 maturation time for cases in which protein abundance is low and readouts are noisy.

### Experimental comparison of different tandem timer designs in developing embryos

**Fig. 2.**
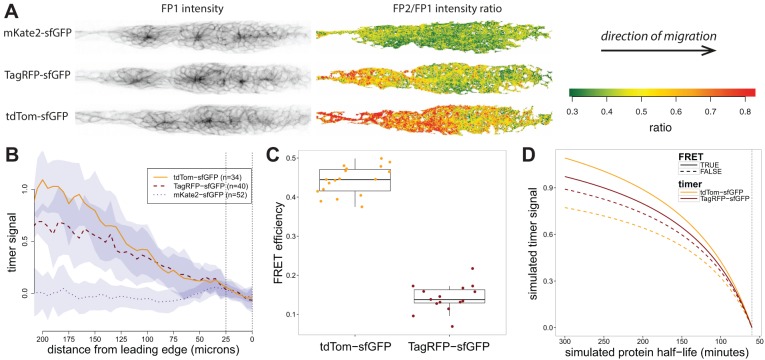
**Timer signal and detection of a chemokine signalling gradient.** (A) Images showing Cxcr4b abundance (left, sfGFP channel) and timer ratios (right) in zebrafish posterior lateral line primordia. Three different timers result in three different ratio profiles. (B) Timer signal along the anterior-posterior axis of the primordium is shown for three timers with different FP2 maturation times. Lines indicate the median ratio across samples and shaded areas the median absolute deviation. In order of profile slope, the timers are mKate2-sfGFP (*n*=52), TagRFP-sfGFP (*n*=40) and tdTom-sfGFP (*n*=34). (C) Estimates of FRET efficiency for tdTom-sfGFP (*n*=14) and TagRFP-sfGFP (*n*=15). (D) Model solutions with and without the experimentally determined FRET efficiencies. The maturation times used were 6 min for sfGFP, 80 min for tdTomato and 100 min for TagRFP. Cxcr4b half-life was assumed to be 1 h at the leading edge of the primordium.

We recently applied tandem timers to demonstrate that a self-generated chemokine signalling gradient guides the migration of the posterior lateral line primordium during zebrafish development. Using the TagRFP-sfGFP timer this was evident as a turnover gradient of the chemokine receptor Cxcr4b across the tissue (Donà et al., 2013). To test the model prediction that increasing FP2 maturation time increases timer signal we reinvestigated this Cxcr4b turnover gradient using three timers (mKate2-sfGFP, tdTom-sfGFP and TagRFP-sfGFP) (Fig. 2A), and generated transgenic zebrafish lines by BAC-mediated complementation. Using the fast-maturing sfGFP as FP1 [maturation time ∼6 min (Khmelinskii et al., 2012)] allowed us to focus on the choice of FP2. The tested FP2s were chosen to have a wide range of maturation times: <20 min for mKate2 (Shcherbo et al., 2009), 60 min for tdTomato (Shaner et al., 2004) and 100 min for TagRFP (Merzlyak et al., 2007). Timer signal was calculated by dividing each ratio profile by its average ratio in the leading 25 μm of the primordium (corresponding to *R^A^* in the model) before summarising across samples and forming the log_2_ ratio (Fig. 2B). This step corrected for differences between timers due to multiplicative effects such as FP2 fluorophore brightness, allowing for direct comparison between timer profiles.

Consistent with model predictions, the timer with the fastest maturing FP2, mKate2, showed a flat profile across the primordium, suggesting that mKate2 timer signal is insufficient to detect differences in Cxcr4b turnover across the tissue. Contrary to model predictions without Förster resonance energy transfer (FRET), the steepest gradient was observed for tdTom-sfGFP despite tdTomato having a shorter reported maturation time than TagRFP. A possible explanation for this discrepancy is that the maturation times of the chosen RFPs in the zebrafish embryo might deviate from previously reported values. However, we assessed this in early-stage embryos via injection of timer mRNAs, and found that the fluorophore maturation time ordering was consistent with reported values (Fig. S1).

Next, we considered FRET. Since the fluorophores are in close proximity, a proportion of sfGFP energy may be absorbed by the RFP, thereby increasing the timer ratio. Incorporation of FRET into the model showed that timer signal increases with FRET (Fig. S2A). This can be understood from the fact that the longer-lived protein pool has a higher proportion of mature FP2 fluorophores available as FRET acceptors. We proceeded to measure FRET efficiencies for tdTom-sfGFP and TagRFP-sfGFP using an acceptor photobleaching approach, and found that the median FRET efficiency between sfGFP and tdTomato was higher than between sfGFP and TagRFP (0.42 versus 0.17; see Fig. 2C). The measured FRET efficiencies proved to be sufficient to explain the experimentally observed ordering when entered into the model (Fig. 2D). These results show that because FRET increases timer signal, it might be a desirable feature for timer design. On the other hand, the presence of FRET complicates the use of FP1 intensity as a direct measure of protein abundance. To assess how FRET might affect the predictions of Fig. 1, we simulated timer signal CV (as in Fig. 1F) as a function of FP2 maturation time and FRET efficiency, and found that FRET has a negligible effect on the optimal choice of FP2 maturation time (Fig. S2B).

### Interpreting tandem timer snapshot measurements in developing embryos

If timer fluorescence and ratio readouts are steady over time, a single snapshot measurement of each fluorescence channel is sufficient to obtain a measure of protein turnover. To take advantage of this capability, users should check that their readouts are close to a steady state, i.e. that they are approximately constant over time. One relevant consideration for developmental processes is the time it takes for timer readouts to attain a steady state. We quantified this from model solutions of FP2 intensity (red lines in Fig. 1B), using the procedure outlined in Fig. S3A. We found that the time to reach steady-state levels, which depends on FP2 maturation time and protein half-life only, can greatly exceed the FP2 maturation time for long-lived proteins (Fig. S3B). Biologists interested in observing rapid processes are therefore advised to use a fast-maturing FP2. If steady-state fluorescence levels cannot be attained, timer ratio comparisons between age-matched samples are still highly informative. If the effects of experimental perturbations are being addressed, care must be taken to control for other factors that may change protein population age, such as differences in developmental timing, protein synthesis kinetics or cell division rate. Further discussion of relevant experimental factors can be found in Khmelinskii and Knop (2014).

### Using tandem timers for investigating degradation and gene expression kinetics

To date, tandem timers have been applied to contexts where changes in protein turnover provided a useful proxy for activity. However, as tandem timers report on protein population age, they also have great potential as reporters of gene expression dynamics in embryonic models. We used computer simulations to explore how time-lapse imaging of timers can inform on expression and degradation kinetics.

**Fig. 3.**
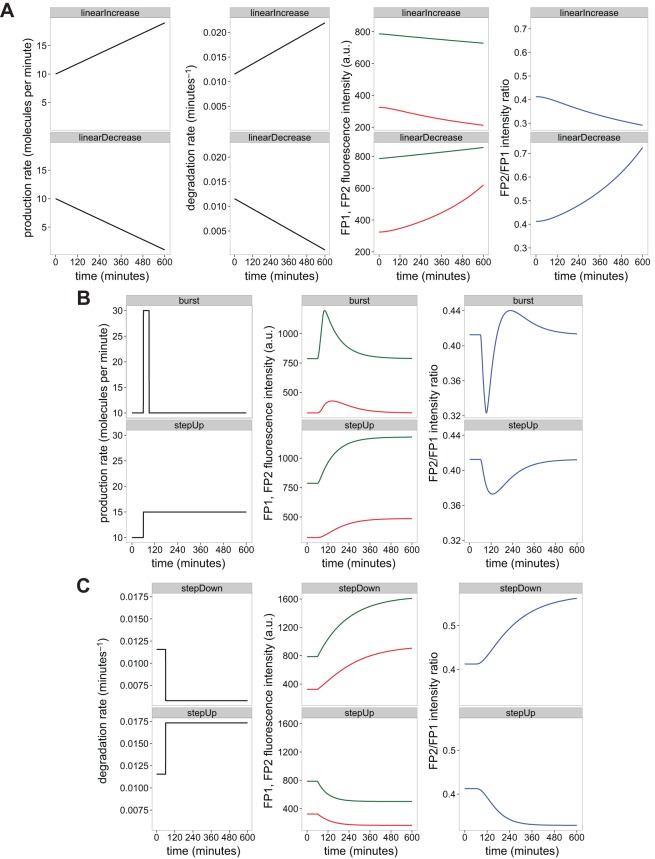
**Using tandem timers for investigating expression and degradation kinetics.** Characteristic models for production and degradation dynamics were solved using steady-state values for FP1 and FP2 populations at time zero. Initial production rates were set to ten molecules per minute. Initial degradation rates were set to log(2)/60 min^−1^. FP1 (green) and FP2 (red) maturation times were set to 5 and 100 min, respectively. (A) Production and degradation rates were set to linearly increase and decrease at comparable rates. (B) Degradation rate was held constant and production rate was increased as a burst and a step up. (C) Production rate was held constant and degradation rate was decreased as a step down and a step up.

First, we investigated the scenario in which proteins were expressed and degraded at comparable rates (Fig. 3A). This highlighted the limitations of using single-colour reporters such as GFP for investigating dynamics, since although FP1 intensity showed approximately constant protein abundance, the FP2/FP1 ratio revealed comparatively dramatic changes in the underlying production and degradation kinetics.

Second, we checked if tandem timers could be used to detect changes in expression kinetics by holding degradation constant and rapidly changing production rate (Fig. 3B). This can be achieved experimentally by expressing a non-degradable version of the timer under identical transcriptional control and localisation as the protein under investigation, as performed previously (Donà et al., 2013). We found that the timer ratio produced a marked response to production changes, and noted that the slower response of the FP2 channel relative to the FP1 channel provided a transient memory that was apparent in the ratio readout. Thus, if turnover can be experimentally fixed by using non-degradable versions of the timer, the ratio provides a reliable readout of gene expression kinetics.

Third, we performed simulations in which production was held steady but degradation rate was rapidly increased or decreased over time, a condition often encountered in response to the activation of signalling cascades (Fig. 3C). The FP1 intensity and FP2/FP1 ratio readouts clearly distinguished between both cases, providing quantitative support for the idea that tandem timers are suitable for investigating processes where rapid changes in protein stability are indicative of biological activity.

Finally, we checked that none of the simulation profile shapes shown in Fig. 3 is affected by FRET (Fig. S4). In summary, tandem timers can be designed to provide reliable readouts for protein degradation and gene expression, making them versatile tools for investigating developmental processes.

### Conclusions

We have modelled timer signal and variability, demonstrating that the choice of the timer fluorophore pairing is crucial for effective detection of protein stability differences in experiments. We experimentally validated predictions of the model using a number of chemokine reporter designs in developing zebrafish embryos, and found that FRET increases timer signal. Recommendations were provided for using the timer in developmental contexts that undergo rapid change. Finally, we used the model to show that timers may be used to study protein degradation and expression kinetics. Software and interactive web applications (http://chronos.embl.de/TimerQuant) are supplied to assist experimentalists in timer design and the interpretation of readouts.

## MATERIALS AND METHODS

### Mathematical methods

The one-step model for fluorophore maturation from Khmelinskii et al. (2012) was used as a basis for all of the derivations and simulations described in this paper (see Mathematical Derivations and accompanying Mathematica notebook in the supplementary Materials and Methods). Timer signal was modelled with the additive noise model:
(1)
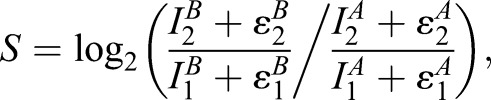
where 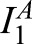, 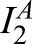, 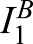 and 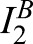 denote fluorescence intensities, the subscripts 1 and 2 denote the FP1 and FP2 fluorescence channels, the superscripts A and B denote the shorter-lived and longer-lived proteins, respectively, and 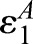, 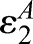, 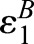, 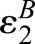
∼*N*(0,*σ*^2^) are independent. Calculations to determine the optimal choice of FP2 maturation time for low abundance proteins were performed in R. Simulations investigating timer responses to expression and degradation kinetics were performed with the R package deSolve (Soetaert et al., 2010). Code reproducing figure panels is provided in the vignette of the Bioconductor (Huber et al., 2015) package TimerQuant.

### Zebrafish husbandry and transgenic lines

Zebrafish (*Danio rerio*) strains were maintained as described (Westerfield, 2007). Embryos were raised in E3 buffer at 30°C. All experiments were conducted on embryos younger than 3 dpf under the rules of the European Molecular Biology Laboratory and the guidelines of the European Commission, Directive 2010/63/EU. Zebrafish strains used in this study included wild-type TU and WIK strains, previously described *cxcr4b:NLS-tdTomato* and *cxcr4b:cxcr4b-TagRFP-sfGFP* (Donà et al., 2013) transgenics, and newly established transgenic lines expressing timer-tagged Cxcr4b: *cxcr4b:cxcr4b-tdTom-sfGFP* and *cxcr4b:cxcr4b-mKate2-sfGFP*. These were generated through BAC-mediated complementation as previously described (Donà et al., 2013). The primers used for amplification of selectable targeting cassettes encoding tdTom-sfGFP and mKate2-sfGFP are listed in Table S1.

### Zebrafish imaging

Imaging and analysis of primordia expressing timer-tagged Cxcr4b reporter were performed on 32 hpf embryos as previously described (Donà et al., 2013). All imaged embryos were heterozygous for the timer-tagged *cxcr4b* transgenic construct.

To obtain fluorophore maturation curves, embryos injected with mRNA encoding the fluorescent timers were imaged on a Zeiss Lightsheet Z.1 microscope for up to 13 h. FRET efficiency measurements were obtained from acceptor photobleaching experiments performed on mRNA-injected embryos. For further information, including details of fluorophore maturation curves, FRET efficiency measurements and plasmids used, see the supplementary Materials and Methods and Table S2.
